# The interplay between exosomal miRNAs and cytokine networks in metabolic diseases

**DOI:** 10.3389/fcell.2026.1731121

**Published:** 2026-02-19

**Authors:** Radhika Joshi, Kanika Verma, Devesh U. Kapoor, Vipin Saini, Swapnil Sharma

**Affiliations:** 1 Department of Pharmacy, Banasthali Vidyapith, Banasthali, Rajasthan, India; 2 Department of Bioscience & Biotechnology, Banasthali Vidyapith, Banasthali, Rajasthan, India; 3 Department of Internal Medicine, Division of Cardiology, LSU Health Sciences Center, Shreveport, LA, United States; 4 Dr. Dayaram Patel Pharmacy College, Bardoli, Gujarat, India; 5 Centre for Research Impact & Outcome, Chitkara College of Pharmacy, Chitkara University, Rajpura, Punjab, India; 6 MM College of Pharmacy, Maharishi Markandeshwar University Campus, Haryana, India

**Keywords:** bioengineering, biomarkers, exosomal miRNAs, metabolic regulation, therapeutic delivery

## Abstract

Exosomal microRNAs (miRNAs) are increasingly recognized as central regulators of metabolic pathways, influencing glucose homeostasis, lipid metabolism, and inflammatory responses. Their ability to mediate inter-organ communication positions them as both biomarkers and therapeutic candidates for metabolic diseases such as type 2 diabetes, obesity, and non-alcoholic fatty liver disease (NAFLD). This review synthesizes recent literature on the mechanistic roles of exosomal miRNAs in metabolic regulation, their influence on insulin sensitivity and lipid homeostasis, and their interaction with cytokine networks. It also evaluates current approaches in exosome engineering, including miRNA enrichment, surface modification, and CRISPR/Cas9-based editing, as well as emerging high-throughput technologies for profiling exosomal miRNAs. Exosomal miRNAs modulate insulin signaling, lipid catabolism, adipogenesis, and inflammatory responses, thereby contributing to metabolic adaptation and disease progression. Specific miRNAs, such as miR-122, miR-155, and miR-34a, have been implicated in obesity, diabetes, and NAFLD, serving as both biomarkers and therapeutic targets. Advances in exosome engineering and delivery strategies have demonstrated improved specificity, stability, and therapeutic efficacy. High-throughput sequencing and single-vesicle analyses provide insights into the heterogeneity and dynamics of exosomal miRNAs, supporting their clinical translation. Exosomal miRNAs represent promising tools for diagnosis and therapy in metabolic disorders. Their dual role as regulators of metabolic processes and carriers of therapeutic cargo underscores their potential in precision medicine. Future integration of bioengineering, CRISPR-based modulation, and omics-driven predictive modeling will enhance their translational applicability, paving the way for personalized therapies in metabolic diseases.

## Graphical Abstract

**Figure:**
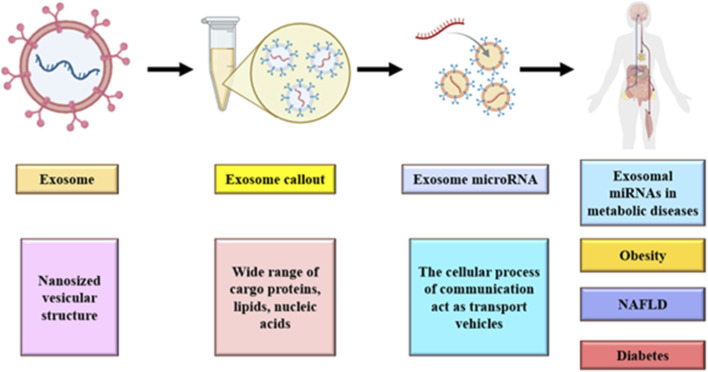

## 1 Introduction

### 1.1 Exosomes as nanocarriers and regulators of metabolic homeostasis

Amongst extracellular vesicles, exosomes are a subgroup of nanovesicles with sizes between 30 and 150 nm, originated from the endosomal pathway through the inward budding of multivesicular bodies, while microvesicles with sizes ranging from 100 to 1,000 nm arise from the direct plasma membrane shedding (Zenatelli, 2023). These vesicles are produced under both physiological and pathological conditions and have emerged as promising nanocarriers owing to their ability to evade rapid clearance, enhance systemic stability, and deliver therapeutic molecules across biological barriers, including the blood-brain barrier (Di Bella, 2022). Exosomes possess inherent safety and can be engineered for loading with nucleic acids, proteins, or synthetic molecules. The surface modification extends the circulation time and target specificity (Liu and Su, 2019). Advanced imaging techniques allow for *in vivo* tracking of exosome biodistribution and pharmacokinetics. Beyond their role as delivery vehicles, exosomes play a critical role in metabolic regulation through their cargo, especially miRNAs (Di Rocco et al., 2016). Exosomal miRNAs mediate intercellular communication by modulating gene expression via 3′UTR binding, leading to mRNA degradation or translational repression, which is essential in maintaining glycolipid and energy homeostasis (Adeli, 2011). These miRNAs influence insulin sensitivity, glucose metabolism, and lipid regulation in metabolic tissues and could modulate macrophage phenotypes, systemic inflammation, and lipid synthesis (Ying et al., 2017). Notably, miR-223, miR-155, and miR-34a control inflammation and macrophage polarization, with impacts on lipid metabolism, while miR-802, miR-99b, and miR-144 influence insulin signaling and secretion, highlighting their potential as biomarkers and therapeutic targets (Amine et al., 2025). In the context of GDM, there are differentially expressed exosomal miRNAs such as miR-423-5p, miR-122-5p, miR-148a-3p, miR-192-5p, and miR-99a-5p. miR-423-5p is upregulated and regulates insulin-related genes such as IGF1R and GYS1, which affect insulin signaling and glycogen synthesis (Ye et al., 2022). On the other hand, miR-122-5p targets G6PC3 and FDFT1, involved in insulin and AMPK signaling pathways. These miRNAs, which can be detected in plasma exosomes well before standard diagnostic windows, are therefore proposed as early biomarkers for gestational diabetes mellitus (GDM) and have been suggested as a signature indicating altered tissue crosstalk among skeletal muscle, liver, adipose tissue, and pancreas that contribute to metabolic dysregulation in diabetes and obesity (Angelescu et al., 2022). Collectively, exosomes function not only as efficient nanocarriers for therapeutics but also as key mediators of metabolic homeostasis, pointing to their dual potential in disease monitoring and treatment (Wang et al., 2024). The Table 1 illustrates the CRISPR derived methodologies for the modulation of mRNA. The Table 2 depicts the different mRNA applications, target pathways for different metabolic disease.

**TABLE 1:** CRISPR-derived methodologies for the modulation of microRNA and the engineering of exosomes in therapeutic contexts.

Exosomal miRNA	Target pathway	Metabolic disease	CRISPR/CAS9 technique	Therapeutic role	References
miR-122	Lipid metabolism and 3-Hydroxy-3-Methylglutaryl-Coenzyme A Reductase (HMGCR)	NAFLD	Gene editing to modulate lipid pathway	Potential for using exosome delivery CRISPR/CAS 9 targeting miR-122 for lipid regulation	Alvares (2023)
miR-29a	Insulin signaling, PI3K/Akt pathway	Type 2 diabetes mellitus	CRISPR-based gene editing for PI3K/Akt	Possible use in editing related genes to improve insulin sensitivity via miRNA regulation	Bhat et al. (2023)
miR-33	Cholesterol metabolism, ABCA1	Atherosclerosis	Exosome-mediated delivery of CRISPR/Cas9	miR-33 inhibition combined with CRISPR editing in cholesterol efflux pathways	ESOBI (2022)
miR-375	Pancreatic β-cell function	Type 2 diabetes mellitus	Gene correction for β-cell preservation	CRISPR based strategies targeting β-cell dysfunction pathways	Domínguez-Bendala et al. (2023)
miR-21	Fibrosis and inflammation	NAFLD, obesity	CRISPR/Cas9 for anti-inflammatory pathways	Potential for modifying pro-inflammatory pathways via miR-21inhibition using CRISPR	Dehghan et al. (2022)

**TABLE 2:** Application of different miRNA, target pathways for diverse metabolic disease.

Exosomal miRNA	Associated metabolic disease	Biological function	Diagnostic/Therapeutic role	References
miR-122	NAFLD	Regulates hepatocyte metabolism	Lipid biomarker for liver damage and dysregulation	Hu et al. (2021)
miR-375	Diabetes	Regulates pancreatic beta-cell function and insulin secretion	Potential therapeutic target for improving beta-cell survival	Latreille et al. (2015)
miR-29a	Type 2 diabetes	Regulates glucose homeostasis and insulin sensitivity	Biomarker for insulin resistance	Massart et al. (2017)
miR-21	Obesity, insulin resistance	Involved in inflammation and adipogenesis	Biomarker for obesity-related inflammation	Cai et al. (2023)
miR-33a/b	Atherosclerosis, obesity	Regulates cholesterol efflux and lipid metabolism	Potential therapeutic target for lipid management	Ortega et al. (2023)
miR-223	Metabolic syndrome	Modulates inflammation and macrophage polarization	Biomarker for systemic inflammation in metabolic diseases	Zhuang et al. (2012)

**FIGURE 1:**
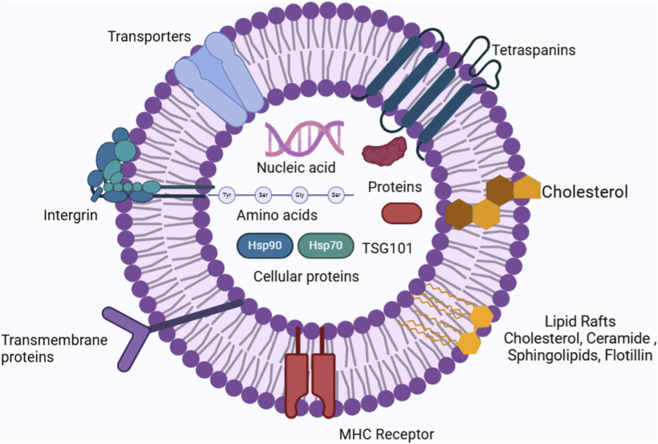
Biochemical composition of exosome carrying several moieties such as nucleic acids and cellular proteins.

Figure 1 illustrates biochemical composition of exosome carrying several moieties such as nucleic acids and cellular proteins.

## 2 Mechanism of exosome miRNA in metabolic regulation

### 2.1 Inter-organ communication and metabolic adaptation

**FIGURE 2:**
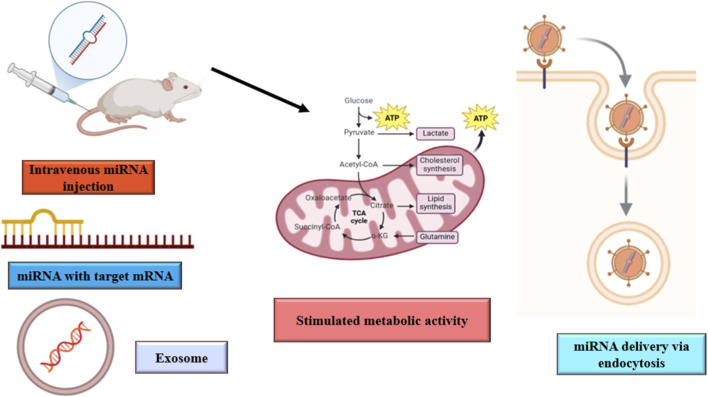
Illustrates a proposed mechanism by which intravenously administered miRNA-loaded exosomes may modulate metabolic pathways in recipient cells.

Exosomal miRNAs act as crucial intercellular messengers, which convey communication between different organs. These are involved in numerous physiological processes, such as immune regulation and trophic support, vital for maintaining metabolic balance (Maia et al., 2018). These miRNAs are transported through the bloodstream into the exosomes, and thus, can be carried to distant organs. The transport mechanism is vital for inter-organ communication, enabling the miRNAs to exert their regulatory effects on metabolic processes in target tissues (Sluijter et al., 2014). Exosomal miRNAs are essential in modulating key metabolic pathways by gene expression modifications in recipient cells. Among the most essential functions performed by exosomal miRNAs is metabolic adaptation through process regulation, especially glycolipid metabolism for optimal energy homeostasis, and crossing through biological barriers to interact with tissues (Isaac et al., 2021). Exosomal miRNAs can transiently interact with the plasma membrane of endothelial cells during circulation (Mantel et al., 2016). This interaction is critical for their uptake and subsequent influence on metabolic functions in various organs, thereby making them a critical component in systemic metabolic regulation (Desideri et al., 2021). The dynamics of exosome-mediated communication *in vivo* were studied using zebrafish embryos as a model. The researchers made use of a fluorescent reporter, CD63-pHluorin, to image the release and trafficking of exosomes from the yolk syncytial layer into the bloodstream, making possible the real-time tracking of these vesicles in the organism. Tracking of exosomes has shown that exosome biogenesis is a syntenin-dependent process, through which they are released into the blood flow, where they move towards distant organs. Once these exosomes have achieved their destination sites, they are engulfed into the cells of macrophages and endothelial. This endocytic process is mediated by scavenger receptors, which is dependent on dynamin. These exosomes later result in the breakdown to occur within lysosomes. This mechanism by which exosomes could transport their content, including proteins, lipids, and RNA, into recipient cells, affects cell functions and plays a part in inter-organ communication. The research also suggests the function of exosomes in promoting vasculogenesis because interference with their production hindered the growth of the CVP, and therefore it may play a trophic role in tissue development. This implies that exosomes act as mediators of communication between organs in the provision of essential signals or materials that support the development and maintenance of tissues (Verweij et al., 2019). Exosomal miRNAs, derived from adipose tissue, play a significant role in inter-organ communication and metabolic adaptation. These miRNAs are important mediators of metabolic homeostasis through endocrine and paracrine mechanisms (Huang and Xu, 2021). Adipose tissue significantly contributes to the circulating pool of exosomal miRNAs, which modulate various metabolic processes in different organs. miR-200 released by adipocytes can be taken up by cardiomyocytes to bring about hypertrophic changes that reflect the inter-organ crosstalk that exosomal miRNAs help to facilitate (Zhang et al., 2019a). More examples of such signaling pathways would include miR-16, miR-27a, miR-146b, and miR-222 released from adipocytes to enter small adipocytes, stimulate them to undergo further differentiation to mature adipocytes, demonstrating paracrine signaling (Aghaei and Hosseini, 2024). Adipose tissue-derived exosomal miRNAs regulate insulin sensitivity and glucose metabolism. The overexpression of miR-155 in adipose tissue macrophage-derived exosomes suppresses insulin action by reducing peroxisome proliferator-activated receptor gamma (PPARg) mRNA, which plays a critical role in metabolic regulation (Sandoval-Bórquez et al., 2024). Transplantation of adipose tissue to adipose-tissue-specific knockout of the miRNA-processing enzyme dicer (ADicerKO) mice was found to enhance glucose tolerance and restore the levels of most exosomal miRNAs, which are part of metabolic adaptation. Studies have indicated that exosome miR-99b is involved in the process of regulating fibroblast growth factor-21 (FGF21), a factor that contributes to metabolic processes, suggesting that exosomal miRNA plays a significant role in metabolic adaptation. The transferred miRNA is miR-16 from muscle exosomes to β-cells, which results in the downregulation of the target gene protein patched homolog 1 (Ptch1). The increased proliferation of insulin-secreting cells was seen in mice that were treated with lipid-enriched diet, suggesting a role of miR-16 in insulin resistance compensation by promoting β-cell proliferation. Moreover, miR-130b is released from adipose tissue, and its presence has been suggested to regulate the function of other metabolic organs. Enhanced circulating concentrations of miR-130b in obese human and murine subjects revealed an equivalent positive association with body mass index. The increased circulating levels of TGF-β in obese mice confirm that release of miR-130b is stimulated by the fact that it is involved in complications related to diabetes (Thomou et al., 2017). These miRNAs, among others, get selectively sorted into exosomes, which are then released into circulation, thus making them possible mediators in communication between different organs. These selective sorting and release mechanisms are also influenced by environmental and pathophysiological conditions to modify the cargo of miRNA in exosomes (Fleshner and Crane, 2017). Therefore, altogether, it would be examples like the two miRNAs, namely, miR-16 and miR-130b, to illustrate the elaborate role that exosomal miRNAs play during inter-organ communication with metabolic adaptation (Kumar et al., 2017). Figure 2 depicts the mechanism by which intravenously administered miRNA-loaded exosomes target specific mRNAs to enhance metabolic activity. These exosomes enter cells via endocytosis, resulting in the modulation of metabolic pathways, including the regulation of glycolysis, the TCA cycle, and the biosynthesis of lipids and cholesterol, ultimately leading to increased ATP production.

### 2.2 Metabolic pathway modulation by exosomal miRNA

Modulation of exosome miRNA in metabolic pathways has a complex mechanism involving oxidative stress and inflammation, key components in the pathogenesis of metabolic diseases (Włodarski et al., 2020). For metabolic diseases, miRNAs in exosomes play a significant role in regulating oxidative stress and inflammation. For example, miR-155, is highly enriched in exosomes isolated from adipose tissue macrophages in mice under an obesogenic diet and has been linked to insulin resistance and glucose intolerance (Duisenbek et al., 2023). This implies that miR-155 may be transported into insulin target cells via exosomes and thereby influence the sensitivity of insulin to contribute to the metabolic effects of obesity. The level of miR-155 in exosomes could be a potential therapeutic strategy to enhance insulin sensitivity and glucose tolerance in metabolic disorders associated with obesity (Kim and Kim, 2021). Moreover, therapies such as administration of pioglitazone have been reported to regulate the expression of certain miRNAs in circulating exosomes. miRNAs like EV-miR-374b-5p and EV-miR-195-5p were lowered in circulating exosomes when pioglitazone was administered, and this showed the contrary in adipose tissue since miR-195-5p was significantly overexpressed. These results indicate that pioglitazone might differentially regulate miRNA expression between tissues, thus modifying the metabolic pathways and possibly restoring insulin sensitivity by down-regulating lipolysis in adipose tissue (Duisenbek et al., 2023).

Several specific non-coding RNAs (ncRNAs) play very important roles in exosome-mediated inter-organ communication. These include miRNAs long ncRNAs, and circular RNAs (circRNAs), which are inside exosomes, enabling such inter-organ communication to occur between adipose tissue and other related tissues for obesity and metabolic diseases (Song et al., 2024). miRNAs comprise small ncRNAs capable of regulating gene expression after transcription. The role played by exosomal miRNAs is crucial in managing the differentiation of adipocytes and lipid metabolism (Brandao et al., 2017). miR-130a-3p secreted from the liver improves glucose intolerance by inhibiting the expression of PHLPP2 in adipocytes, showing that this small RNA has metabolic regulation (Wu et al., 2020). lncRNAs are long ncRNAs, capable of regulating gene expression at various levels including chromatin modification, transcription, and post-transcriptional processing. lncRNAs are long ncRNAs, having the potential to modulate gene expression at numerous levels such as chromatin modification, transcription, and post-transcriptional processing. These are covalently closed-loop non-coding RNAs (circRNAs), and hence are stable and exonuclease-resistant (Taylor et al., 2015). Exosomal circRNAs are emerging as important regulators in cell-to-cell communication, influencing various physiological and pathological conditions, including those related to obesity and metabolic diseases (Ashrafizadeh et al., 2022).

Exosomal miRNAs are important in the regulation of metabolic pathways, particularly in obesity and obesity-related metabolic disorders. The miRNAs are selectively secreted by cells and packaged in exosomes, which are small vesicles that enable intercellular communication (Isaac et al., 2021). In the study, it was noted that the exosomal miRNA profile significantly changes in obesity, and some miRNAs were more abundant in obese than in lean individuals. Among the miRNAs found were miR-192 and miR-122, both of which were overexpressed in obesity. The researcher has been described as being overexpressed in prediabetes and insulin resistance conditions, thus implicated in the first steps of metabolic syndrome development. The researchers showed that exosomes transfected with synthetic mimics of the miRNAs found highly represented in obese mice induced glucose intolerance, adipose inflammation, and hepatic steatosis in lean mice upon systemic injection. This implies that these miRNAs can modulate glucose and lipid metabolism, hence the metabolic changes (Castaño et al., 2018).

The mechanistic pathway involves the delivery of these miRNAs by exosomes to the target cells, where it could cause transcriptomic alterations. This process affects the body’s sensitivity to insulin and contributes to glucose intolerance and dyslipidemia (Agbu and Carthew, 2021). The study further highlighted that the pattern of miRNA in obese exosomes was distinct enough to be differentiated from that of lean exosomes, emphasizing its potential as a biomarker for metabolic disorders. More importantly, the worsening of glucose intolerance and insulin resistance when high–fat diet (HFD) was added in exosome-treated mice indicates that these miRNAs are potentially very significant in affecting metabolic pathways, independent of obesity itself (Castaño et al., 2018).

### 2.3 Influence of exosomal cargo on cellular energy balance

Exosomes transport a wide array of bioactive cargo, including proteins, lipids, and nucleic acids, each of which contributes to regulate cellular metabolism and intercellular communication (Donoso-Quezada et al., 2021). Exosomal proteins represent enzymes, signaling mediators, and structural components participating in key metabolic pathways such as glucose utilization, lipid handling, and insulin signaling, thus influencing metabolic homeostasis and disease progression (Wei et al., 2021). Some exosomal proteins regulate intracellular signaling cascades involved in cell growth, differentiation, and cellular stress responses-processes increasingly recognized as shared regulatory features across metabolic and inflammatory disorders (Corrado et al., 2013). The lipids of exosomes are derived mostly from the plasma membrane of donor cells and, by influencing vesicle stability and membrane fluidity, modulate receptor-mediated signaling in recipient cells. These lipid components play important roles in immune activation and inflammatory signaling, which are central to the pathophysiology of obesity, insulin resistance, and metabolic syndrome (Chimal-Vega et al., 2025). In parallel, exosomes shuttle functional nucleic acids, including mRNAs and microRNAs, that reprogram gene expression in target tissues. Exosomal mRNAs can be translated in recipient cells, whereas microRNAs fine-tune metabolic pathways by suppressing genes involved in insulin signaling, lipid metabolism, and inflammatory regulation, thereby directly contributing to metabolic disease development (Guay and Regazzi, 2017). Beyond a mere molecular transfer, exosomal microRNAs are increasingly recognized as active regulators of cellular energy balance and metabolic adaptation. Through modulation of pathways that govern nutrient sensing, mitochondrial activity (Deng et al., 2024), and cytokine signaling, exosomal microRNAs impact tissue remodeling, immune-metabolic crosstalk, and systemic energy distribution (Mansoori et al., 2022). These mechanisms are particularly relevant in metabolic disorders, where chronic inflammation, vascular remodeling, and immune cell activation impose sustained energetic stress on tissues such as adipose depots, liver, and skeletal muscle.

### 2.4 Interplay between exosomal miRNAs and cytokine networks

The exosomal microRNAs serve as major mediators in intercellular communication, fine-tuning cytokine responses as they are transferred from metabolic and immune cells. There are some known exosomal miRNAs that regulate inflammatory cytokine expression by specifically targeting major signaling factors; this then affects macrophage polarization as well as events in systemic metabolic inflammation (Fernández-Messina et al., 2015). A good case in point in this concern was given by exosomal miR-155, a major component of exosomes from macrophages that drive these immune cells towards a pro-inflammatory phenotype. The miR-155 directly targets SOCS1, a natural regulator that suppresses the JAK/STAT pathway; this increases the secretion of pro-inflammatory cytokines TNF-α, IL-6, and IL-1β and further triggers insulin resistance in metabolic organs (Maciak et al., 2021). Conversely, miR-146a in exosomes is known to have anti-inflammatory properties. Since miR-146a targets IRAK1 and TRAF6, it inhibits the activation of NF-κB and reduces the production of inflammatory cytokines like TNF-α and IL-6, but facilitates the polarization of M2-like macrophages and thus plays a role in maintaining metabolic homeostasis (Ding et al., 2021). The next critical regulator is miR-21 in exosomes, which associates metabolic stress with cytokine responses. By targeting PTEN, miR-21 activates the PI3K/AKT signaling pathway and stimulates the production of IL-10, along with influencing pro-inflammatory responses in macrophages and adipocytes, particularly in obesity (Melnik et al., 2020). miR-122-enriched exosomes, originating from the liver and targeting PPARα and regulators of the metabolic pathway, may affect the interaction between the immune and metabolic systems in the targeted macrophages. In this process, the production of pro-inflammatory cytokines such as IL-6 and TNF-α is increased, thus establishing the link between lipid metabolism and inflammation in the liver during metabolic disorders (Brigstock, 2021). miR-34a exosomal functions are pivotal in the regulation of M2, an anti-inflammatory morphotype defined by the transcription factor KLF4, whose suppression is induced by increased miR-34a expression, causing low levels of the anti-inflammatory factor IL-10 and increased production of pro-inflammatory cytokines, hence propagating inflammation associated with obesity (Carvalho-Paulo et al., 2021).

## 3 Exosome engineering for metabolic disease therapy

### 3.1 Strategies for miRNA enrichment and exosome loading

There are different methods to load exosomal miRNA in exosomes that impart specific characteristic to exosomes. This is carried out by an incubation process whereby mixtures of miRNAs and exosomes are under specific conditions for the enhancement of the loading process (Xi et al., 2021). Cholesterol-modified miRNAs are reported to have more loading efficiency through increased stability, and faster attachment to the cell membranes that enhance their internalization to the cell, thereby providing enhanced silencing of gene expression (Uddin et al., 2023). Sonication is a loading method that involves the use of sound waves to temporarily open a hole in the exosome membrane to allow entry of miRNAs (Liu et al., 2024). Transfection is another loading method, whereby donor cells are transfected with plasmids encoding the desired miRNA, which then becomes encapsulated into exosomes during their biogenesis (Yang et al., 2020b). This method can attain very high loading efficiency and stability but introduces contaminants from transfection reagents. This is a strategy in which donor cells are co-incubated with miRNAs, allowing the natural cellular processes to encapsulate the miRNAs into exosomes (Yang et al., 2024). Electroporation involves applying a temporary electrical field that would open the pores of the exosomal membrane and, therefore allow the miRNA to penetrate inside (Morshedi Rad et al., 2021). Despite the wide use of miRNA loading approaches such as electroporation, sonication, or transfection, several technical nuances exist that might complicate the interpretation of loading efficiency and function (Pottash, 2022). For example, electroporation might induce RNA aggregation, destabilization of the vesicle membrane, sonication might induce aggregation of exosomal vesicles, while transfection might result in the presence of contaminants or RNA exosomal complexes (Choi et al., 2024). To avoid the complications in interpreting the role of exosomes using these approaches, several control measurements need to be considered (Syn et al., 2017). They include protecting the preparation with RNase/protease assays to validate the presence of the RNA in the vesicles, use of spike RNA controls for normalization purposes, while mock electroporation or mock transfection protocols would help distinguish exosomal from non-exosomal effects (Cao et al., 2024). Although several promising strategies are available, this emerging technology still requires further development and refinement. Several challenges still need to be addressed, including limiting cargo leakage from endogenous sources, possible aggregations of exosomes and miRNAs that impede functionality of nucleic acids, presenr in the exosomes (Askenase, 2021). Some strategies that have been used to overcome these limitations include, the physical method, through the use of calcium chloride (CaCl_2_) with heat shock. The addition of CaCl_2_ facilitates the uptake of miRNA by enhancing the interactions between miRNAs and the exosomal surface (Mousaei Ghasroldasht et al., 2024). Heat shock further increases the fluidity of the exosomal membrane, thus making entry easier for miRNA. The enrichment of exosomes with particular miRNAs and their proper loading involves several critical steps (Corbeil and Lorico, 2020). Another strategy such as the application of an electrical field to increase the membrane permeability of the exosome also facilitates the entry of desired miRNA. Specifically, the electroporation is done for characterization of exosomes and to confirm the successful loading of desired miRNA-155. For the internalization of these miRNA-155-loaded exosomes by dendritic cells (DCs), the exosomes were pre-exposed at a concentration of 100 μg/mL. The exosome transfer into DCs was followed through fluorescence microscopy and quantitative real-time PCR to ascertain that the exosomes were indeed being internalized by the DCs (Asadirad et al., 2022).

### 3.2 Bioengineering exosomes; functional enhancement for targeted delivery

Bioengineering exosomes to enhance their performance and directed delivery requires numerous sophisticated mechanisms to improve therapeutic potential (Villata et al., 2020). The ultimate objectives of bioengineering exosomes are their prolongation of circulation, half-life, and increasing the presence of bioactive cargos in the vicinity of the desired target location (Mondal et al., 2023). These effects can be achieved by applying two broad approaches: manipulating before or after isolation. Pre-isolation manipulation of parent cells from which the exosomes are derived may be a part of that. This can be achieved through direct treatment of the cells with a specific drug or cargo (Li et al., 2017). There has been emerging research indicating the need for release methods based on devices with long-lasting release mechanisms, using a combination of extracellular vesicle delivery and molecular analysis on the device itself, to sufficiently deal with rapid EV removal rates (Verma et al., 2025). Alternatively, it can also be achieved through genetic alterations using plasmids bearing miRNA or siRNA. These modifications ensure the exosomes produced carry desired therapeutic agents or genetic materials that can influence the target cells behavior (Mondal et al., 2023). Finally, post-isolation manipulation involves altering the exosomes after they have been obtained from the parent cells. This can be achieved through physical or chemical methods and can incorporate additional therapeutic agents or enhance the stability and targeting capabilities of exosomes (Taylor and Shah, 2015). These modifications are necessary to ensure that the exosomes can deliver their cargos effectively to the target site of injury, maximizing their therapeutic impact. Genetic manipulation of parent cells to present functional peptides on the exosome surface (Liang et al., 2021a). This is done by fusing peptides to membrane proteins of exosomes, such as tetraspanins, although these proteins are not broadly used due to their internal N and C-terminals, which complicate fusion without disrupting protein function (Goo et al., 2024). Another approach is using bio-orthogonal chemistry that allows the introduction of non-native moieties on the exosome surface. This technique is advantageous since it allows the inclusion of a wide variety of targeting ligands, which, in turn, enhances the ability of the exosome to home in on certain cells or tissues (Aljoudi et al., 2021). Direct modification to isolated exosomes also offers advantages, such as that abundant and diverse ligands can be incorporated, and this would improve the *in vivo* biodistribution and targeting efficiency of exosomes. Surface modification by physical interactions including charge interaction and hydrogen bonding is another strategy (He et al., 2024). Compared with chemical reactions, weaker and reversible bonds are achieved, but shorter bond-formation time and higher efficiency of bonding are obtained (Baek et al., 2015). This allows target-specific moieties to encompass the exosomal surface, using a negative charge that is the result of different membrane proteins. The streptavidin conjugation to plasma membrane proteins of exosomes, this can be done by creating a chimera between streptavidin and signal peptide and transmembrane segments, enabling the streptavidin-biotin system to be used for decorating the exosome surface (Ferreira et al., 2022). This method allows for many biotinylated molecules like peptides and antibodies to bind to the surface of the exosomes; thus, the targeting ability can be increased. One, conjugated biotinylated peptides and antibodies attached exosomes can target selected cell or tissue-specific uses, and this gives great convenience in the handling, development, and therapy utilization using nano-therapy method due to the availability of such versatile modification systems and enables the modification of an exosome to affect not more than one disease (Li et al., 2024). The hybridization of synthetic liposomes with cellular membrane-derived vesicles (CMVs). This hybrid system increases the yield and stability of the vesicles but also allows for donor cell surface proteins to be integrated. These proteins could potentially bind to specific receptors on target cells, hence enhancing the precision of active targeting for therapeutic agent delivery (Jiang et al., 2022). Another approach is to synthesize core/shell nanostructures, where synthetic nanoparticles act as the core and cell membrane structures as the shell. This design does not only stabilizes the nanoparticles but also retains their natural features, such as size and shape, which play an important role in passing through physiological barriers (Zhou et al., 2024). The cell membrane shell also endows a biomimetic surface that can evade immune detection and enhance targeting toward specific tissues or cells. CMV/scaffold complexes are used to achieve long-term release of therapeutic agents. The structures of scaffolds support CMVs that ensure controlled, long-term release of the drugs thereby extending their *in vivo* bioavailability. It is therefore beneficial in cases where there is a need to prolong the therapeutic level of the drugs over extended periods to reduce the administration frequency, thus improving compliance among patients (Jang et al., 2023).

### 3.3 CRISPR/CAS 9 tools for precision miRNA modulation in exosomes

Exosomes, being biocompatible and capable of crossing biological barriers, are the ideal delivery vehicle for CRISPR/Cas9. It can carry the CRISPR/Cas9 components to specific cells, where the guide RNA (gRNA) guides the Cas9 nuclease toward the target miRNA sequences (Zhang et al., 2024a). Targeted delivery is important to ensure that the CRISPR/Cas9 system modulates the miRNA expression in the desired cells. On arriving at the target cells, the CRISPR/Cas9 system triggers a double-strand break in the specific miRNA loci (Chen et al., 2023). This would either knock out the miRNA or modify its expression and thus modulate it. The accuracy of the process is essential for effective outcomes in therapy, to reduce the rate of cell proliferation and migration in cancerous cells (Momen-Heravi et al., 2014). CRISPR/Cas9 has greatly helped in generating animal models that recapitulate the genetic and phenotypic features of diabetes. Lep and Lepr gene-targeted knockout mouse models have been established, which recapitulate the diabetic phenotype, characterized by increased body weight and hyperglycemia (Bora et al., 2023). These models are critical to understanding the molecular mechanisms of diabetes and to test therapeutic interventions. The CRISPR/Cas9 system has been used to introduce gene-editing tools directly into adipose tissues; these tools target genes relevant to obesity and metabolic syndrome. The use of the CRISPR interference system (CRISPRi) was observed to silence the Fabp4 gene, reduce inflammatory responses in adipose tissue, and thus alleviate both obesity and hepatic steatosis. This strategy underlines the prospect of treating obesity-related metabolic disorders by CRISPR/Cas9. Obesity is intimately associated with Type 2 Diabetes Mellitus (Wang et al., 2022). Here, using the gene target of interest in macrophages such as netrin-1, the administration of CRISPR/Cas9 has resulted in the enhancement of glucose tolerance and improved insulin sensitivity in diabetic mice. This is due to the modulation of macrophage activity in adipose tissues, which significantly contributes to the inflammatory processes involved with insulin resistance. In the case of Type 1 Diabetes Mellitus (T1DM), CRISPR/Cas9 is utilized for the deletion of the Renalase (RNLS) gene, whose interference has a significant role in modulating immune cell responses. Deletion in this context leads to minimizing the autoimmune attack on the pancreatic beta cells, preserving insulin production and functioning. Such interventions are important in the management of autoimmune aspects of T1DM (Dehghan et al., 2022). Off-target effects result in unintended genetic alterations, potentially causing adverse outcomes. Immune activation is another concern, as the delivery of CRISPR components can trigger immune responses that affect safety and efficacy (Lopes and Prasad, 2024). Efficient and targeted delivery to specific tissues remains a major limitation, particularly *in vivo*. Additionally, regulatory frameworks for genome-editing therapeutics are still evolving, posing challenges for clinical approval (Han et al., 2020). Addressing these factors is essential to ensure the safe and effective application of CRISPR/Cas9 technologies in human therapies. Zhang et al. demonstrated that an exosome-derived long non-coding RNA (AK083884) released from M2 macrophages confers protection against CVB3-induced viral myocarditis in mice by modulating macrophage metabolic reprogramming via the PKM2/HIF-1α signaling axis (Zhang et al., 2024b).

## 4 Therapeutic applications of exosomal miRNAs in metabolic diseases

### 4.1 Diabetes mellitus; exosomal miRNAs as regulators of insulin sensitivity

In the context of diabetes, the pathological environment associated with the disease influences the biosynthesis of exosomes and the sorting of miRNAs within peripheral tissues exhibiting insulin resistance, thereby modulating the expression profiles of exosomal miRNAs. A number of specific miRNAs have been recognized as key modulators of insulin sensitivity within the disease framework (Chang and Wang, 2019). Specifically, miR-20b is significantly overexpressed in skeletal muscles under diabetic conditions, indicating it plays a role in insulin sensitivity modulation in this tissue. In the liver, miR-15b overexpression acts on insulin receptors, contributing to liver insulin resistance, an important factor in the pathogenesis of diabetes (Kaur et al., 2020). miR-143 expression is significantly upregulated in the liver of dietary obese mice, further implicating its role in insulin resistance. The liver also preferentially packages miR-122 into microvesicles, resulting in elevated serum and reduced liver content in type 2 diabetic mice, thus implicating the microRNA in lipid metabolism and insulin sensitivity (Liang et al., 2024).

miRNA-802 is crucial in controlling glucose transport. This is a microRNA that would be considered a potential drug target for type 2 diabetes because of its contribution to insulin sensitivity and the mechanisms of glucose transport (Zhen et al., 2018). In skeletal muscle cells, expression is significantly increased in conditions with high lipids that are often associated with diabetes. Upregulation suggests miR-802 contribute to impaired glucose transport that occurs in diabetic states by altering insulin signaling pathways (Zhang et al., 2023a). Increased expression in obese mice models and humans makes it a possible mediator in glucose metabolism and insulin resistance. Through the modulation of gene expression in these pathways, miR-802 can contribute to the metabolic dysfunctions characteristic of diabetes, and thus it is a critical factor in disease progression and a potential target for therapeutic intervention (Włodarski et al., 2020). The miRNAs, including miR-27a, miR-34a, miR-141-3p, miR-155, miR-210, and miR-222 are packaged in extracellular vesicles from adipose tissue and other origins. miR-27a affects the insulin signaling pathway by targeting genes that regulate glucose metabolism. miR-34a modulates pathways of insulin signaling and thereby plays a role in glucose homeostasis (Payet et al., 2024). miR-141-3p affects the insulin sensitivity pathway by targeting components of the insulin signaling pathway, and miR-155 is associated with inflammatory responses, affecting insulin sensitivity through inflammation-related gene expression (Chatterjee et al., 2024). miR-210 is implicated in metabolism regulation, and it affects insulin sensitivity by disrupting energy metabolism and mitochondrial functioning (Geiger and Dalgaard, 2017). miR-222 regulates insulin sensitivity by downregulating genes that correspond to cell cycle and apoptosis pathways. The imbalance of such miRNAs leads to insulin resistance, a factor of diabetes mellitus; therefore, these miRNAs are considered therapeutic targets with the potential to manage and improve insulin sensitivity in people with diabetes mellitus (Chien et al., 2015). miR-27a levels elevate, thus enhancing insulin resistance. This miRNA suppresses the expression of the PPARγ, a transcription factor, which regulates genes involved in lipid and glucose metabolism thereby impairing insulin sensitization (Chen et al., 2019). Furthermore, miR-27a impacts the PPARγ/PI3K/Akt/GLUT4 signaling pathway, which is crucial for glucose uptake in cells, further contributing to insulin resistance. It also leads to inflammation by promoting the infiltration and activation of macrophages through PPARγ inhibition and NF-κB-mediated transcription (Chen et al., 2012). microRNA-27a exerts its regulatory effects on mitogen-activated protein kinase 14, a key player in the translocation of GLUT4 through the insulin receptor substrate 1/Akt signaling pathway; consequently, it modulates glucose uptake in both skeletal muscle cells and adipose tissue. These actions combined demonstrate how miR-27a disrupts normal glucose metabolism, contributing to the development of insulin resistance during obesity (Li et al., 2022). Exosomal miRNA-148a has been shown to suppress AMPK signaling and is significantly present in high levels within milk. Suppression leads to an increase in the levels of mTORC1, which can cause de-differentiation in β cells, mediate endoplasmic reticulum (ER) stress, and impair the capacity of β cells for insulin secretion. This process is critical because the appropriate function and maturation of β cells depend on the switch from mTORC1 to AMPK signaling (Melnik, 2019). The existence of exosomal miRNA-148a prevents such a switch, thus preventing the maturation and proper functioning of β cells, which is essential for maintaining regular insulin levels as well as preventing the development of type II diabetes (TIID) (Entezari et al., 2022). The exosomal miRNAs present in milk also have been implicated in TIID development by influencing mTORC1 and AMPK signaling pathways. In this regard, dietary influences, such as milk intake, may modulate diabetes molecular mechanisms through the exosomal miRNA regulation of molecular mechanisms. Interestingly, treatments including bacterial fermentation, boiling, or ultra-heat treatment of milk can suppress and degrade these exosomal miRNAs, potentially preventing the development of TIID (Kakoti et al., 2024).

**FIGURE 3:**
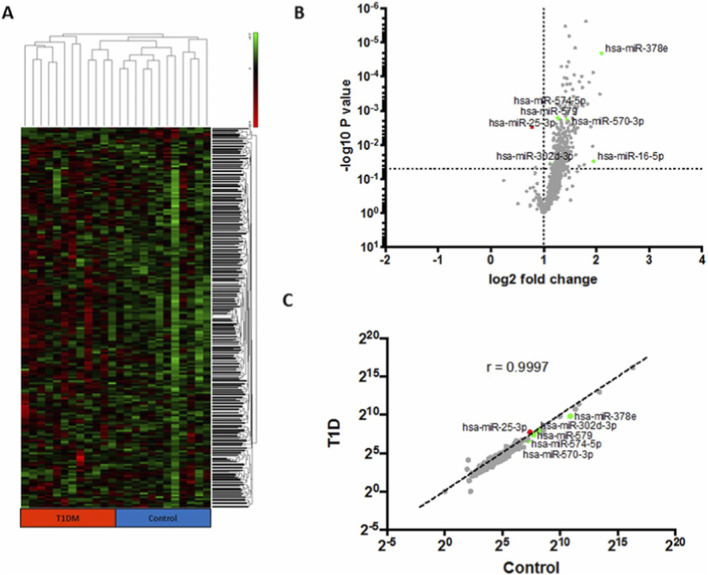
**(A)** Heatmap of per-row normalized expression levels of selected miRNAs differentially expressed in plasma-derived exosomes originating from T1DM and control subjects. **(B)** Scatter plot showing miRNAs enriched in control (green) or T1DM (red) subjects, n = 12 per group. Multiple t-test, p < 0.05. **(C)** Correlation plot of exosomal miRNAs, using red and green coloration for indicating miRNAs differentially expressed between T1DM and control samples. [Adapted with permission from Garcia-Contreras et al. (2017)].

Garcia-Contreras et al. conducted a study in which Plasma-derived exosomes were isolated from T1DM patients and normal controls by differential centrifugation, and their identity was verified by nanoparticle tracking analysis and transmission electron microscopy. Total miRNA profiling of exosomal RNA from 12 T1DM and 12 control subjects was performed using the Nanostring human v2 miRNA microarray, followed by bioinformatic analysis using the nSolver software. Seven miRNAs were found to be significantly dysregulated in T1DM-derived exosomes, including one upregulated miRNA, miR-25-3p, and six downregulated miRNAs, namely, miR16, miR-302d-3p, miR-378e, miR-570-3p, miR-574-5p, and miR-579. These candidate miRNAs were further validated by qRT-PCR in expanded cohorts, 24 T1DM and 24 controls, which confirmed their consistent deregulation. More importantly, most of these miRNAs have been reported to be involved in immune regulation, β-cell survival, and inflammatory signaling pathways relevant to T1DM pathogenesis. The changed exosomal miRNA signature in T1DM not only underscores their diagnostic values but also suggests their potential therapeutic efficacies because exosome-mediated delivery of regulatory miRNAs be used to modulate autoimmune responses and preserve β-cell function. Collectively, this study provides mechanistic insight into the exosome-based miRNA communication in T1DM and supports their utility as a novel class of therapeutic targets or delivery vehicles. The comparative profiling of plasma-derived exosomal miRNAs in T1DM and control revealed distinct expression patterns between the two groups. As shown by hierarchical clustering analysis, samples were clearly segregated based on differentially expressed miRNAs (Figure 3A). Subsequent Scatter plot analysis identified miRNAs significantly enriched in either control or T1DM subjects, underscoring one upregulated and six downregulated miRNAs in T1DM (Figure 3B). Moreover, correlation analysis of exosomal miRNAs disclosed coordinated expression changes associated with T1DM pathophysiology, which underscores their functional and therapeutic relevance (Figure 3C). Differential miRNA content in plasma-derived exosomes from T1DM and control subjects (Garcia-Contreras et al., 2017).

### 4.2 Obesity and lipid dysregulation; targeting adipose and liver function

**FIGURE 4:**
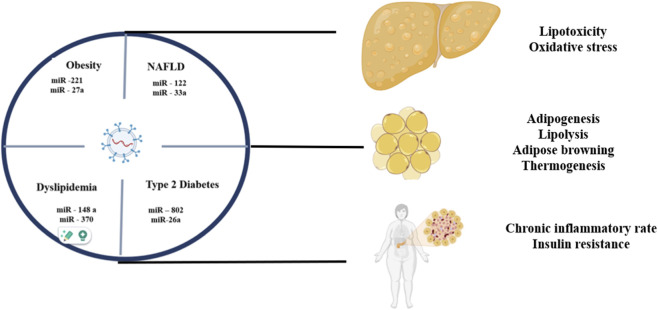
A schematic depiction elucidating the involvement of particular microRNAs in metabolic pathologies associated with obesity, non-alcoholic fatty liver disease (NAFLD), dyslipidemia, and type 2 diabetes, mediated by mechanisms such as lipotoxicity, adipogenesis, and insulin resistance.

The role in the regulation of lipid metabolism and obesity exosomal miRNAs exert by the alteration in lipid catabolism, storage, and lipogenesis. Exosomal miRNAs in the setting of obesity may influence several pathways for gene expressions thereby altering the lipid metabolism process (Hegde et al., 2023). Hepatocyte-derived exosomal miRNA-34a is recognized to repress key genes involved in lipid oxidation, such as peroxisome proliferator-activated receptor alpha (PPARα) and carnitine palmitoyltransferase II (CPTII), in which fatty acid oxidation (FAO) and lipolysis are necessary (Hochreuter et al., 2022). Exosomal microRNAs associated with obesity have been correlated with the dysregulation of mitochondrial lipid oxidation and fatty acid metabolic pathways, thereby influencing the expression of peroxisome proliferator-activated receptor alpha (PPARα) and peroxisome proliferator-activated receptor gamma coactivator 1-alpha (PGC1-α) genes (Kaur et al., 2015). The deregulation may lead to disrupted lipid catabolism, thus worsening obesity-related metabolic dysfunctions. In terms of lipid storage, exosomal miRNAs modulate lipid deposition in tissue (Maligianni et al., 2021). miRNA-34a is indicated to correlate with increased amounts of visceral fat and inflammatory markers in obesity-related metabolic dysfunction, which states its function in lipid storage and inflammation (Pan et al., 2019). miRNA 19b has been involved in reducing gestational obesity by modulating inflammatory cytokines and lipid droplet formation, pointing out its function regarding lipid storage and inflammation (Zaiou et al., 2018). miRNAs can directly or indirectly interfere with the activities of important enzymes in the process of lipogenesis. They can alter the expressions of acetyl-CoA carboxylase and fatty acid synthase; these are enzymes that are essential in the process of producing fatty acids (Yu et al., 2019). They intervene in the regulatory pathways in control of the signaling involved in lipogenesis. They can influence pathways, for example, the insulin signaling pathway, wherein activation of downstream transcription factors leads to increased expression of lipogenic genes, thus promoting lipogenesis (Guan and Chen, 2014). miRNAs that originate from adipose tissue could promote hypertrophy, the enlargement of adipocytes, and, because larger adipocytes require more space to store lipids, there is a close correlation between the increase of lipogenesis and hypertrophy (Youssef et al., 2020). miR-122 is known to be one of the most abundant in the liver and is said to modulate lipid metabolism. miR-122 affects the levels of genes involved in both fatty acid and cholesterol synthesis, thus directly taking a part in lipogenesis (Wei et al., 2016). miR-33 plays an important role in cholesterol as well as fatty acid metabolic regulation. The molecule seems to interfere with genes needed for the synthesis and storage of lipids; it points towards important genes like sterol-regulating element binding proteins (SREBP), as it inhibits the development of new adipocytes along with several key transcription factors that orchestrate lipogenesis in miR-27 (Price and Fernández-Hernando, 2015; Yu et al., 2018). miR-103/107 have been reported to play roles in regulating insulin sensitivity and lipid metabolism by influencing genes of interest that participate in the mechanisms of lipogenesis (Joven et al., 2012). Figure 4 demonstrates schematic illustration highlighting the role of specific microRNAs in metabolic disorders (Zhang et al., 2025) linked to obesity, non-alcoholic fatty liver disease (NAFLD), dyslipidemia, and type 2 diabetes (Ren et al., 2019), through pathways involving lipotoxicity, adipogenesis, and insulin resistance.

**FIGURE 5:**
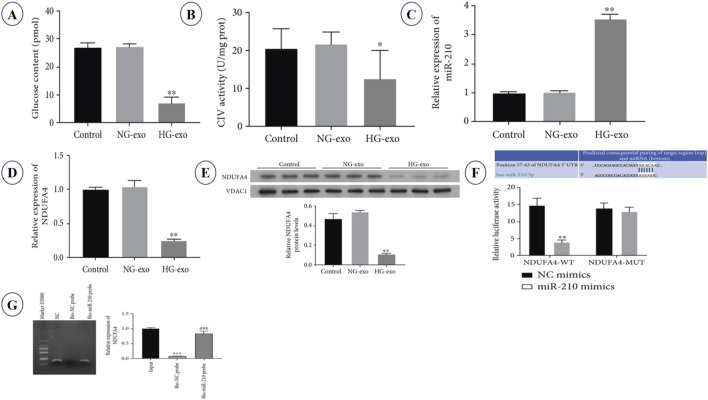
Macrophage-derived exosomal miR-210 suppresses glucose uptake of adipocyte and mitochondrial complex IV activity through targeting NDUFA4. **(A)** Glucose uptake in 3T3-L1 adipocytes treated with exosomes derived from RAW264.7 macrophage cultured under normal-glucose (NG-exo) or high-glucose (HG-exo) conditions. **(B)** Mitochondrial respiratory chain complex IV (CIV) activity in 3T3-L1 adipocytes treated with macrophage-derived exosomes, measured by ELISA. **(C)** Relative expression of miR-210 in 3T3-L1 adipocytes after incubation with macrophage-derived exosomes. **(D)** mRNA expression levels of NDUFA4 in 3T3-L1 adipocytes treated with NG-exo or HG-exo. **(E)** The protein expression of NDUFA4 in 3T3-L1 adipocytes was determined by Western blot, in which the VDAC1 protein was used as an internal control. **(F)** Predicted miR-210 binding sites within the 3′UTR of NDUFA4 gene and confirmation of the direct targeting by using dual-luciferase reporter assays with wild-type (WT) and mutant (MUT) constructs. **(G)** The interaction between miR-210 and NDUFA4 mRNA was confirmed in a biotin-labelled miR-210 pull-down assay. [Adapted with permission from Tian et al. (2020)].

Tian et al. (2020) studied that exosomes derived from high glucose-stimulated RAW264.7 macrophages were highly positive for miR-210 and damaged glucose uptake and mitochondrial respiratory chain complex IV (CIV) activities (Figures 5A,B). Construction of 3T3-L1 adipocytes with these exosomes strongly reduced glucose uptake and mitochondrial respiratory chain complex IV (CIV) activities (Figures 5A,B), but increased intracellular miR-210 and decreased the transcription level and protein expression of mitochondrial respiratory chain complex IV component NDUFA4 (Figures 5C–E). Further mechanism studies showed that miR-210 targets the 3′-UTR of NDUFA4 (Figure 5F) and directly targets NDUFA4 by miR pull-down (Figure 5G), establishing an exosomal direct regulatory pathway. Importantly, miR-210 inhibition and genetic deletion reversed glucose uptake and mitochondrial respiratory chain complex IV activities and reduced diabetic obesity (Figures 5A,B) and pathological indexes to normal control levels (Figures 5C–E), but reduced diabetic obesity (Tian et al., 2020).

### 4.3 Non–alcoholic fatty liver disease; modulating inflammatory pathways

Exosomal miRNAs play a crucial role in the regulation of PPARα, which is an essential transcription factor in lipid metabolism. These miRNAs are known to influence PPARα expression by different pathways, thereby regulating lipid breakdown and storage (Hegde et al., 2023). Hepatocyte-derived exosomal miRNA-34a downregulates PPARα, which is essential for fatty acid oxidation and lipolysis. This suppression can lead to lipid accumulation, thus contributing to metabolic disorders like NAFLD (Ma et al., 2024). Obesity-related exosomal miRNAs are associated with deregulated mitochondrial lipid oxidation and fatty acid metabolism and affect PPARα as well as other related genes such as PGC1-α. This kind of deregulation can result in impaired lipid catabolism and further contributes to obesity-related metabolic dysfunctions (Hegde et al., 2023). PPARα is one of the target genes regulated by exosomal miRNA-96-5p, indicating its potential role in lipid uptake and metabolism in the context of diseases, such as non-alcoholic steatohepatitis (NASH) (El-Derany and AbdelHamid, 2021). However, exosomal miRNAs are shown to suppress the expression of PPARα, causing an imbalance in the homeostasis of lipid metabolism and generating disease progression for metabolic disorders (Agbu and Carthew, 2021). As modulators of PPARα and of other pathways, exosomal miRNAs are interesting candidates for therapeutic intervention in the treatment of diseases related to lipid regulation (Liu et al., 2023). Exosomal miRNA-199a-5p has been implicated in modulating lipid accumulation to aggravate NAFLD. This miRNA modulated lipid synthesis gene expression, which contributed to the progression of the disease (Hromadnikova et al., 2022). Bone-marrow mesenchymal stem cells (BMSC) exosomes-derived miRNA-96-5p alleviated NASH, a severe form of NAFLD, through downregulation of genes related to lipogenesis, such as SREB1, SREB2, and acetyl-CoA carboxylase (ACACA). This modulation helps restore lipid homeostasis and might be a therapeutic approach for NASH (Hegde et al., 2023). miRNA-690 was found to be an inhibitor of the NASH phenotype, fibrogenesis, and *de novo* lipogenesis in mice, showing that it takes part in lipid balance maintenance and disease prevention (Cunha e Rocha et al., 2024). The miRNA-106b-5p from the macrophage causes mitochondrial dysfunction and impacts FAO by degrading genes like ACSL4, essential for lipid degradation. This disruption can exacerbate NAFLD by damaging lipid breakdown (El Hayek et al., 2024). miRNA-34a exacerbates inflammation and lipid accumulation in models of NAFLD by inducing inflammatory cytokines and lipogenic genes. This particular miRNAs overexpression is linked with enhanced lipid peroxidation and increased oxidative stress, thereby disrupting lipid homeostasis (Su et al., 2018). miRNA-122-5p accelerates the progression of NASH by inducing polarization of macrophages in M1 and increasing levels of inflammatory cytokines, which subsequently results in increased lipid storage as well as circulation problems (González-Blanco et al., 2024).

**FIGURE 6:**
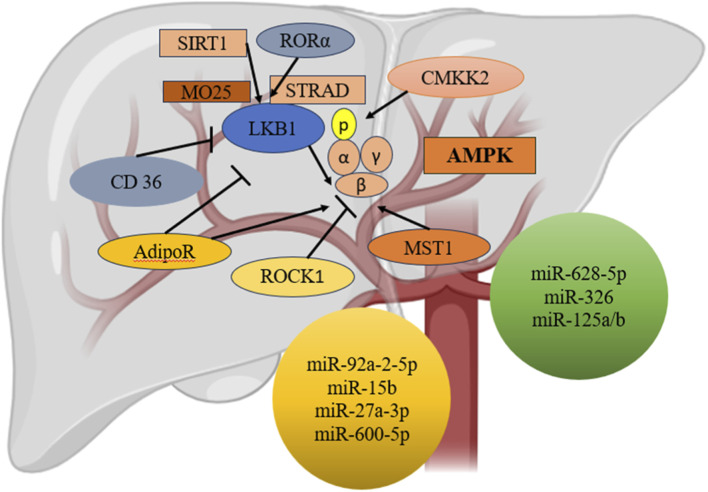
Regulatory framework governing the activation of AMP-activated protein kinase (AMPK) within hepatic tissues. Interactions among pivotal proteins and microRNA signaling pathways that are integral to the maintenance of metabolic equilibrium and the regulation of energy homeostasis.

The overexpression of miR-205-5p is identified as an important event in the pathogenesis of NAFLD. It contributes to enhancing inflammation by supporting macrophage polarization. This disease is associated with central aspects of its pathogenesis involving inflammatory responses, which have been implicated by the action of miR-205-5p. Its mechanism of operation is mediated by the binding action of this miRNA toward the retinoic acid receptor-related orphan receptor α (RORα) (Zhang et al., 2023b). Targeting RORα leads to the modulation of miR-205-5p in the polarization of macrophages towards the M1 phenotype, which is associated with the production of pro-inflammatory cytokines (Tavakoli Pirzaman et al., 2024). The modulation of macrophage polarization by miR-205-5p increases the inflammatory environment within the liver, thus contributing to liver damage and NAFLD progression. Inhibition of miR-205-5p was associated with a reduction in inflammation, suggesting its therapeutic value (Zhou et al., 2022). This mechanistic understanding emphasizes the exosomal miR-205-5ps significance in NAFLD, putting exosomal miRNAs at large, with significant implications regarding diseases of the liver associated with inflammation and immune deregulation. Figure 6 illustrates the regulatory mechanisms controlling the activation of AMP-activated protein kinase (AMPK) in liver tissue, including the interplay of key proteins and microRNA signalling pathways essential for sustaining metabolic balance and managing energy homeostasis.

**FIGURE 7:**
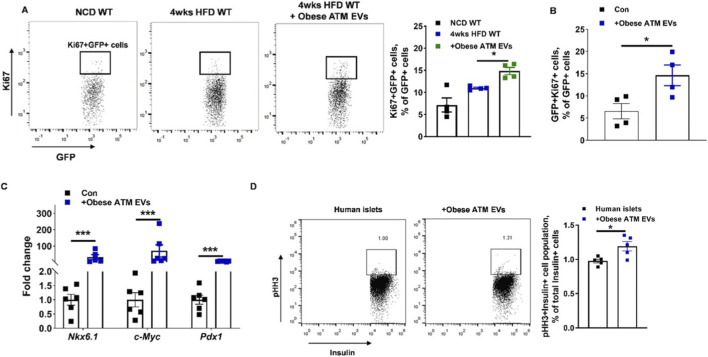
Obese ATM-derived EVs enhance β-cell proliferation in obesity. **(A)** Measurement of proliferating Ki67^+^GFP^+^ β-cells within the islets of MIPGFP-Mki67 mice maintained on NCD, 4-week HFD-fed mice, and HFD-fed mice administered. **(B)** *In vitro* analysis of the proliferation of Ki67^+^GFP^+^ β-cells in NCD MIPGFP-Mki67 islets exposed to EVs derived from **(C)**. Relative expression of genes involved in β-cell mass enhancement after 72 h of obese ATM-derived EV treatment. **(D)** Analysis of proliferating pHH3^+^Insulin^+^ cells in human islets exposed to obese ATM-derived EVs. [Adapted with permission from Gao et al. (2021)].

Gao et al. (2021) studied that EVs released by obese ATMs contain microRNA that acts as an endocrine message targeting pancreatic β cells. Using both *in vivo* and *in vitro* systems, the authors have shown that EVs secreted by obese ATMs inhibit the secretion of insulin but stimulate the growth of β cells, which represents a compensatory increase in the mass of β cells due to the development of insulin resistance. These findings have also been demonstrated in human islets. From a mechanistic perspective, the research proves that the biological activities of obese ATM EVs are miRNA-driven. EVs derived from Dicer-knockout ATMs, which have little to no miRNA, showed negligible effects on the proliferation and insulin secretion of β cells, proving the crucial role of miRNA. Among the different miRNAs identified, it is found that miR-155 is significantly enriched in obese ATM EVs. Overexpression of miR-155 in β cells inhibits insulin secretion, but promotes β cell proliferation. Genetic deletion of miR-155 suppresses the regulatory function of obese ATM EVs. A further study reveals the Mafb protein as the key target for miR-155, proving the crucial function of the miR-155-Mafb axis in the functional adaptation of β cells. Notably, the *in vivo* delivery of obese ATM EVs to HFD-induced MIPGFP-Mki67 mice led to a substantial increase in the percentage of proliferative Ki67-positive β cells (Figure 7A) with no impact on apoptosis but a concomitant decrease in the overall β cell insulin content. Further, obese ATM EVs significantly increased the percentage of proliferative cells within cultured mouse islets *in vitro* (Figure 7B), as well as the expression levels of genes linked with the expansion of the pool of β cells (Figure 7C). These increases due to obese ATM EV exposure were also reflected by the percentage of dividing insulin-positive cells in human islets (Figure 7D) (Gao et al., 2021).

## 5 Safety and immunogenicity of exosome therapeutics

Exosomes are naturally produced in the cellular microenvironment, they are biocompatible by nature which in turn minimizes toxicity and negative immune reactions compared to other synthetic carriers (Zhang et al., 2019b). Patient-derived exosomes autologous in nature have lower immunogenicity risks but are difficult to manufacture; allogenic and cell-derived exosomes are easier and faster to produce but require rigorous testing of immunogenicity and pathogenicity effects (Desai et al., 2023). Although exosomes penetrate immune cells through their lipid bilayer and exhibit cellular mimicry to avoid recognition, their miRNA load, surface changes, or impurities stimulate unfavorable immune reactions (Gurunathan et al., 2021). This remains so because depending on what they contain, exosomes could possess pro or anti-inflammatory properties and specific miRNA loads, and surface markers some of which may mean activation of specific immune pathways such as the toll-like receptors (TLRs) must be fine-tuned (Nag et al., 2023). They have demonstrated tolerability and selective toxicity in animal models in preclinical studies but their human application has limitations which can be tested after formulation and clinical trials to establish dosages, routes of administration, and potential side effects. Engineered exosome miRNA therapeutics will not pose toxicity issues if the miRNA payload is precisely regulated, and their source tissue is of high quality while following production guidelines (Ahn et al., 2022). Going forward, the adoption of uniform assay and reliable regulation goals will remain significant in supporting these therapies as safe and effective thus creating the desired opportunities to become part of clinical practices.

## 6 High-throughput platforms for tracking exosomal miRNA dynamics

High-throughput techniques developed for the analysis of exosomal miRNA dynamics are fundamental in clarifying the complex roles of exosomal miRNAs in intercellular communication, disease progression, and therapeutic strategies (Sharma et al., 2024). These methodological approaches utilize state-of-the-art technologies encompassing next-generation sequencing (NGS), quantitative Polymerase Chain Reaction (qPCR), microarray analysis, and innovative single exosome analytical instruments that provide exceptional sensitivity and specificity. NGS facilitates comprehensive miRNA profiling, revealing not only the quantitative levels but also variations in sequences such as iso-miRNAs, which significantly influence their biological activity (Srivastava, 2018). Microarrays provide a high-throughput and cost-effective approach for exploratory studies involving large cohorts; however, they are limited to analyzing a predefined panel of miRNAs (Juracek et al., 2022). Quantitative PCR, particularly the advanced digital and multiplex versions, has made it possible to accurately quantify particular miRNAs for specific research and the right validation of biomarkers (Morales and Ko, 2022). Single exosomes have only recently been analyzed because novel techniques developed at the level of individual vesicles as droplet digital PCR and nano-flow cytometry, made such analysis feasible (Liang et al., 2021b). This advance is very important because it could enlighten the functional diversity present within the exosomal population, and also enlighten how their cellular origins influence the composition of their miRNA cargo (Ferguson and Nguyen, 2016).Newer isolation methods, such as microfluidics and immune-affinity-based approaches, further surge the purity of exosomal preparations, an essential step to guarantee the integrity of the downstream analyses (Yang et al., 2020a). The combination of microfluidic EVs separation using AI-assisted data analysis improves the accuracy and feasibility of EV-miRNA biomarkers discovery. The combination does this in terms of the purity of the vesicles separated, recognizing patterns, and analyzing data. The combination provides a platform for non-invasive disease diagnosis and further stimulates quality control measures for the rapid translation of diagnostic platforms based on EV-miRNAs (Verma et al., 2025). This integration of high-throughput platforms with bioinformatic methodologies enables extensive analysis of data, and it helps in establishing relationships among miRNA profiles, biological pathways, and clinical outcomes (Perakakis et al., 2018).

**FIGURE 8:**
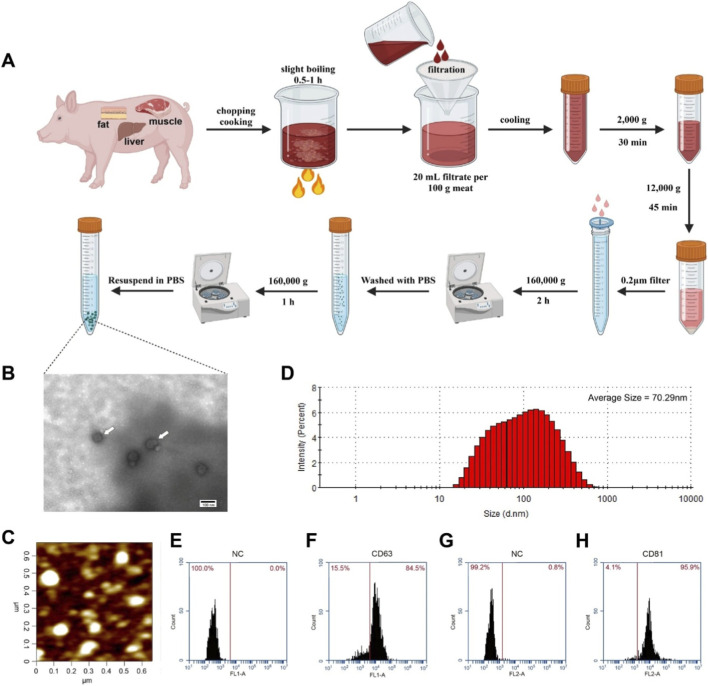
Isolation and characterization of exosomes from cooked meat. Exosomes were successfully isolated from porcine muscle, fat, and liver **(A)** and displayed typical vesicular morphology **(B)** with uniform spherical topology **(C)**. Size distribution analysis confirmed nanoscale dimensions **(D)**, and flow cytometry verified the presence of canonical exosomal markers CD63 and CD81 **(E–H)**, confirming the identity and purity of the isolated vesicles. [Adapted with permission from Shen et al. (2023)].

Shen et al. (2023) showed an integrated high-throughput protocol for isolating, profiling, and functionally tracking exosomal miRNAs from cooked pork tissues. Exosomes were extracted from cooked porcine muscle, fat, and liver by differential ultracentrifugation and purified by sequential filtration and phosphate-buffered saline washes. Their shape and nanoscale integrity were established by transmission electron microscopy (TEM) and atomic force microscopy (AFM), showing spherical vesicles of 20–200 nm. The dynamic light scattering (DLS) measurements revealed a particle size of ∼70 nm, whereas flow cytometric analyses confirmed exosomal nature by high expression of CD63 and CD81 markers on the surface. With next-generation small RNA sequencing, the authors characterized the exosomal miRNA cargo of each tissue type and determined that their content was strongly tissue-specific. Exosomes from muscle tissue were enriched with miR-1, miR-133a-3p, and miR-206, while exosomes from liver tissue contained primarily miR-122, miR-451, and miR-99a. KEGG pathway enrichment analyses for predicted target genes showed strong enrichment for insulin resistance, MAPK, autophagy, and TNF signaling pathways, which indicates potential regulatory roles of these miRNAs in metabolic homeostasis. To assess systemic uptake and physiological effect, ICR mice were treated with exosome-rich drinking water for 80 days chronically. Quantitative RT-PCR validated the substantial increases in important exosomal miRNAs (miR-1, miR-133a-3p, miR-206, and miR-99a) in mouse plasma, accompanied by an everted intestinal sac assay that confirmed gastrointestinal absorption of the vesicles. Following metabolic evaluation with glucose tolerance (GTT) and insulin tolerance (ITT) tests, impaired glucose metabolism as well as insulin sensitivity in the exosome-fed group was demonstrated. Liver histology by HE and Oil Red O staining indicated more lipid droplet deposition, characteristic of hepatic steatosis. High-throughput RNA-seq transcriptome analysis of liver tissues identified 466 differentially expressed genes (292 up- and 174 downregulated) enriched in steroid biosynthesis, cholesterol and lipid metabolism, and MAPK signaling pathways. These integrated multi-omic analyses offered mechanistic insights that dietary exosomal miRNAs are able to modulate host metabolic pathways, supporting the food-borne exosomal communication mechanism in metabolic dysregulation. Overall, this high-level study illustrates how simultaneous nanoparticle characterization, high-throughput sequencing, and transcriptomic integration can be applied to track exosomal miRNA dynamics and downstream consequences in metabolic disease models. Nanoscale exosome characterization combined with high-throughput miRNA sequencing revealed differential miRNA cargo and systemic metabolic effects of cooked-meat-derived exosomes (Figures 8A–H) (Shen et al., 2023).

## 7 Challenges and limitations

The process of translating exosome-based therapies into practical, applicable therapies is hindered by the fractured and rapidly shifting regulatory environment. The regulatory environment varies greatly depending on the region, whether it is the US, the European Union, or Asian countries. In addition to the natural variability found within EVs, the variations cause concern for defining, evaluating, and verifying the efficacy and safety of the products. Conversely, the absence of standardized processes for quality control, such as effective tests for potency, the degree of consistency between batches, and GMP-compliant processes for production, presents barriers for clinical translation. Recent analyses advocate for harmonized and standardized regulation processes and methods, and further suggest policy reforms for overcoming the regulatory disparities across the globe for clinical translation (Verma and Arora, 2025). Although exosomal miRNAs hold great therapeutic promise for the treatment of metabolic diseases, a number of significant challenges and obstacles stand in the way of their clinical applicability. First and foremost is the difficulty of facile and accurate miRNA loading into exosomes, for which electroporation, sonication, and transfection are commonly used techniques that often suffer from such defects as cargo leakage, aggregation of exosomes, and reagent contamination, ultimately losing nucleic acid potency and therapeutic potency. Additionally, the population heterogeneity of exosomes makes reproducible isolation of homogenous subsets of vesicles with uniform bioactive content essential to guarantee therapeutic reliability. Immunogenicity is still a major issue; while exosomes inherently enjoy biocompatibility, their miRNA content, surface antigens, and possible contaminants might elicit unwanted immune reactions, such as activation of toll-like receptor signaling pathways, requiring strenuous safety and immunogenicity assessments. In addition, biodistribution, pharmacokinetics, and controlled release of exosomal miRNAs are still not well understood, hindering accurate dosing and delivery strategies. Although exosome therapies are truly promising, their translation into clinical applications is faced with challenges. It is difficult to manufacture these therapies on a large scale, as it is challenging to produce many exosomes that are uniform in their properties and activities. It is essential to standardize the isolation and purification processes of these exosomes, as variations in these steps may lead to differences in yield, purity, and biological activities. Moreover, their safety, particularly their possible immunogenic and off-target activities, should be investigated comprehensively before undertaking clinical applications. After this stage, adaptability to regulatory frameworks is necessary, given that there are currently no standard guidelines for their characterization and approval as clinical agents.

## 8 Conclusion

Exosomal miRNA modulate crucial pathways including insulin signaling, lipid metabolism, and inflammatory responses that maintain cellular metabolic homeostasis. They play pivotal role during development of insulin resistance and energy metabolism leading to type-2 diabetes, obesity, NAFLD and cardiovascular diseases. In addition, they contribute to inter-organ communication and are considered as potential therapeutic targets. Interestingly, exosomes can be engineered to provide therapeutic outcomes using several bioengineering methodologies, consisting of CRISPR/Cas9-based gene enhancing, miRNA enrichment techniques, and surface functionalization strategies, keeps enhancing the specificity and efficacy of therapeutics derived from exosomes. The arrival of subsequent technology sequencing and single-vesicle analysis has furnished deeper insights into the complexities of exosomal miRNA dynamics, while additionally facilitating the identity of novel biomarkers and the improvement of personalized therapeutic modalities.

## 9 Future perspective

The future of miRNA therapeutics from exosomes in metabolic disorders is to develop novel bioengineering and integrative multi-omics techniques to bypass existing limitations and gain improved clinical efficacy. Progress in genetic and chemical manipulation of parent cells and exosome modification after isolation will enhance the loading efficiency, stability, and tissue-targeting of miRNA cargos such as the application of bio-orthogonal chemistries and hybrid vesicle systems that fuse synthetic and cell membranes for enhanced circulation and targeted delivery. Single-exosome analytical and high-throughput sequencing technologies are expected to untangle the heterogeneity and functional heterogeneity of exosomal miRNAs, thus facilitating personalized therapeutic regimens that target distinctive metabolic pathways involved in diseases like type 2 diabetes, NAFLD, and obesity. CRISPR/Cas9 genome editing provided through exosomes is an innovative route for direct pathogenic miRNA and target gene modulation, potentially allowing for long-lasting and disease-modifying treatments. In addition, the synergy of machine learning and bioinformatics with omics data will enable predictive modeling of inter-organ crosstalk by miRNAs and therapeutic response, which will speed up rational design and regulatory harmonization. With standardized manufacturing protocols and solid safety profiles being confirmed by clinical trials, exosomal miRNAs will emerge as key elements of precision medicine regimens targeting metabolic dysregulation and comorbidities.
